# Recurrent reversible in-stent-stenosis after flow diverter treatment

**DOI:** 10.1007/s00234-023-03144-7

**Published:** 2023-03-28

**Authors:** Malvina Garner, Frederik Fries, Alena Haußmann, Michael Kettner, Armin Bachhuber, Wolfgang Reith, Umut Yilmaz

**Affiliations:** Department of Neuroradiology, Saarland University Hospital, Kirrberger Str, D-66424 Homburg, Germany

**Keywords:** Intracranial aneurysm, Flow diverter stent, In-stent-stenosis

## Abstract

Flow diverter stents (FDS) are well established in the treatment of intracranial aneurysms which are difficult to treat with conventional endovascular techniques. However, they carry a relatively high risk of specific complications compared to conventional stents. A minor but frequent finding is the occurrence of reversible in-stent-stenosis (ISS) that tend to resolve spontaneously over time. Here, we report the case of a patient in their 30s who was treated with FDS for bilateral paraophthalmic internal carotid artery (ICA) aneurysms. ISS were found at the respective early follow-up examinations on both sides and had resolved at the 1-year follow-up examinations. Surprisingly ISS reoccurred at both sides in later follow-up examinations and again resolved spontaneously. The recurrence of ISS after resolution is a finding that has not been described previously. Its incidence and further development should be investigated systematically. This might contribute to our understanding of the mechanisms underlying the effect of FDS.

## Introduction

Reversible in-stent-stenosis (ISS) is a common finding after treatment with flow diverter stents (FDS) [1, 2]. Animal studies suggest that neointimal hyperplasia and mural thrombus formation are mechanisms involved in its genesis [3, 4]. Though the occurrence of ISS after FDS treatment is well described, it has not been reported that ISS reoccurs after initial resolution so far. Here, we report a case of recurrent reversible ISS after FDS treatment of bilateral ICA aneurysms.

## Case presentation

**Fig. 1:**
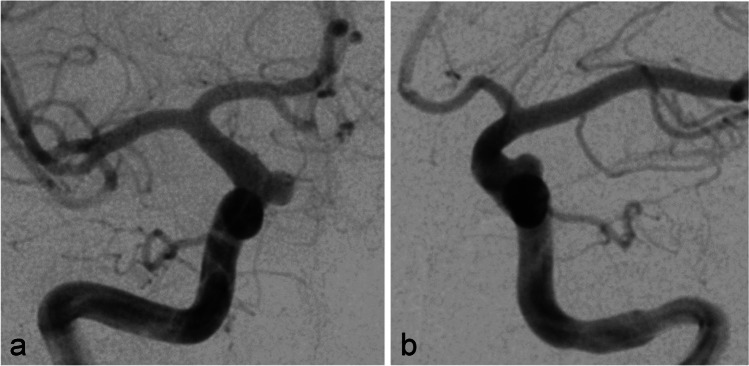
Digital subtraction angiography showing paraophthalmic ICA aneurysms on the right side (**a**) and on the left side (**b**) before FDS treatment

**Fig. 2:**
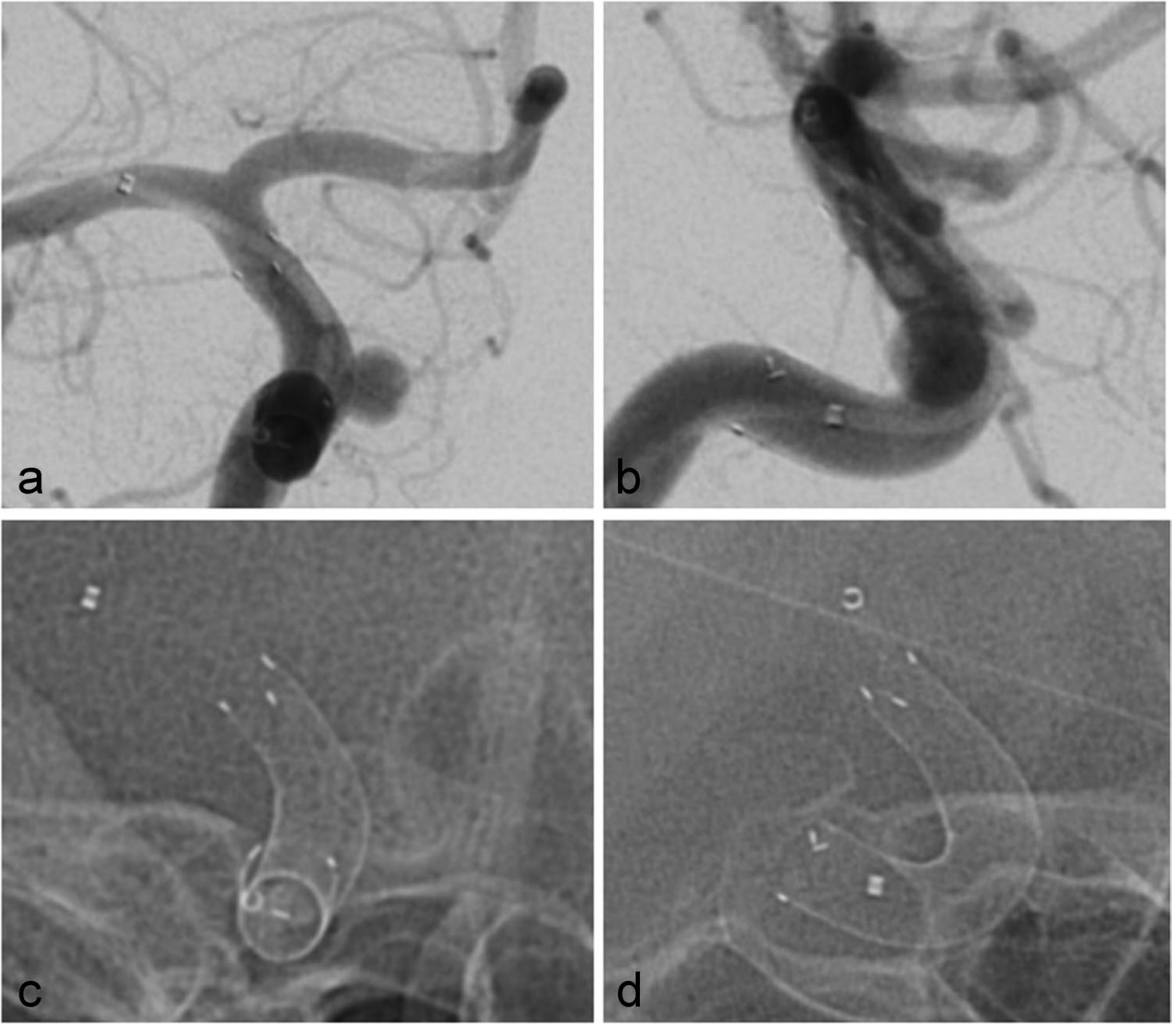
Digital subtraction angiography (**a** and **b**) and non-subtracted images (**c** and **d**) showing width and positioning of the FDS immediately after implantation

**Fig. 3:**
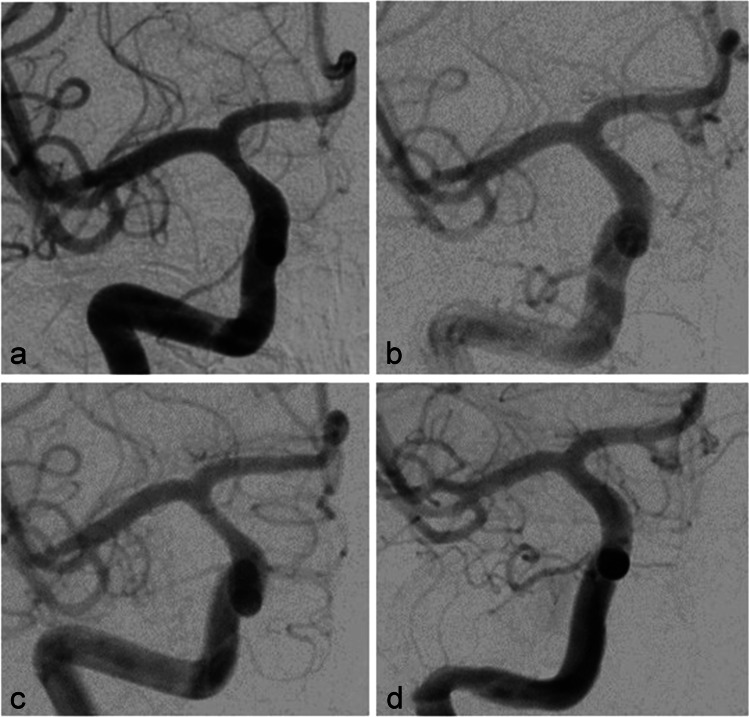
Digital subtraction angiography follow-up examinations of the right ICA showing 50% ISS after 5 months (**a**) and 31 months (**c**) which resolved after 14 months (**b**) and 44 months (**d**), respectively

**Fig. 4:**
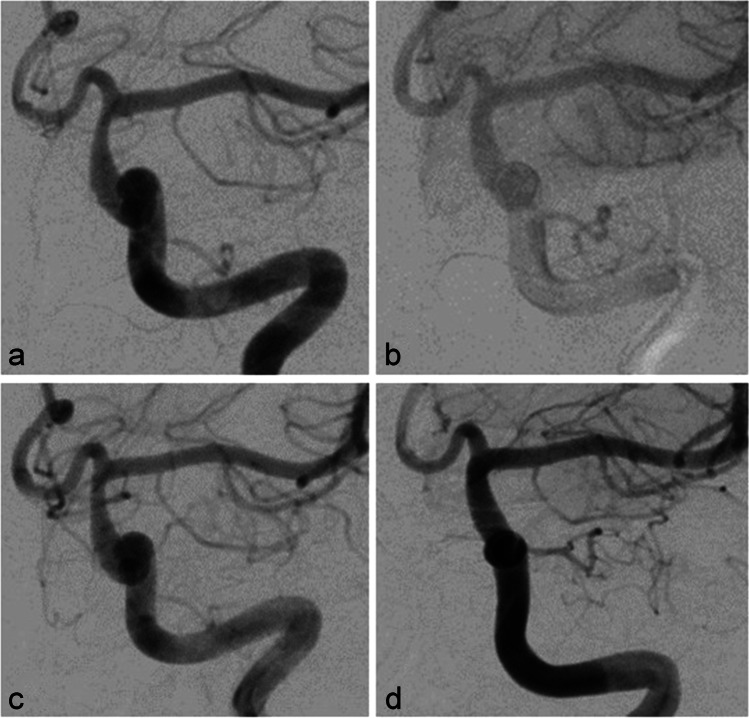
Digital subtraction angiography follow-up examinations of the left ICA showing 60% ISS after 3 months (**a**) and 50% ISS after 29 months (**c**) which resolved after 12 months (**b**) and 42 months (**d**), respectively

A patient in their 30s was administered to our department because of the incidental finding of bilateral asymptomatic paraophthalmic ICA aneurysms. Aneurysms were found on a routine cranial MRI examination that was performed because of chronic headaches. There were no other pre-existing illnesses and no cardiovascular risk factors. The aneurysms were saccular broad-based with a mean diameter of 5 mm on each side (Fig. 1). A dual antiplatelet therapy with ASA 100 mg/day and clopidogrel 75 mg/day was established 3 days before treatment and continued during and after treatment. Platelet function testing before flow diversion is not routinely performed in our department and was not performed in this case either. Treatment with FDS was performed without complications on the right ICA first with a Derivo FDS (Acandis GmbH, Pforzheim, Germany) (Fig. 2) and on the left ICA 2 months later with a Pipeline Flex FDS (Medtronic, Minneapolis, MN, USA). Early follow-up examinations which were performed 5 months after treatment of the right side and 3 months after treatment of the left side respectively showed complete occlusions of both aneurysms and a 50% ISS at the right FDS (Fig. 3a) and a 60% ISS at the left FDS (Fig. 4a). The patient had no symptoms, so no measures other than continuation of the dual antiplatelet therapy were taken. ISS had resolved at the next follow-up examinations that were performed after 14 months and 12 months, respectively (Figs. 3b and 4b). The antiplatelet therapy was reduced to ASA 100 mg/day. Surprisingly ISS reoccurred at the follow-up examination at 31 and 29 months with 50% stenosis on either side (Figs. 3c and 4c) and again resolved at the next follow-up examinations at 44 and 42 months after implantation (Figs. 3d and 4d).

## Discussion

We report a case of recurrent reversible ISS after treatment of intracranial aneurysms with FDS which to our knowledge has not been reported before. Reversible ISS is a well-known minor complication of FDS treatment which has been reported in several studies [1, 2]. It is often found in early follow-up examinations at about 3 months after treatment and has typically resolved in follow-up examinations after 12 months. Animal studies suggest that neointimal hyperplasia and mural thrombus formation are mechanisms involved in its genesis [3, 4].

We can only speculate about the reasons why the recurrence of ISS after initial resolution has not been reported before. First of all, it might be a very rare complication which therefore has not been observed so far. However, a review of our own institutional follow-up routine showed that follow-up examinations of patients treated with FDS are rarely performed by DSA after more than 2 years but are usually performed by MRI. This might contribute to a low rate of detection since MRI with standard sequences has reduced specificity for stenosis in arterial segments treated by FDS [5].

The recurrence of ISS after initial resolution is a new finding that needs further investigation in clinical and experimental studies. Since intimal hyperplasia is responsible for the initial reversible ISS, it is likely that it also causes the recurrence of ISS. However, there is no available data from clinical or experimental studies regarding long-term cellular changes after FDS treatment. Long-term follow-up MRI examinations should therefore include imaging sequences that are sensitive for in-stent-stenosis such as black-blood-imaging sequences [5]. The further investigation of recurrent reversible ISS might contribute to our understanding of the mechanisms underlying the general effects of FDS.
